# A Study on 10-Week Combined Aerobic and Resistance Training Exercise Prescription for Female Patients with Pelvic Floor Dysfunction

**DOI:** 10.3390/healthcare13060592

**Published:** 2025-03-08

**Authors:** Lu Zhang, Jingbo He, Quancheng Zhang, Ling Wang

**Affiliations:** 1 Faculty of Medicine, Macau University of Science and Technology, Macau 999078, China; 3230004406@student.must.edu.mo; 2 College of Mathematics and Computer Science, Tongling University, Tongling 244061, China; jingbohe@tlu.edu.cn; 3 School of Physical Education, Xi’an Shiyou University, Xi’an 710065, China; zhangqcheng@xsyu.edu.cn

**Keywords:** pelvic floor dysfunction, aerobic exercise, resistance training, exercise therapy, postpartum women

## Abstract

**Background/Objectives**: Female pelvic floor dysfunction (FPFD) is a prevalent condition affecting postpartum women. This study aims to evaluate the effectiveness of a 10-week combined aerobic and resistance training exercise prescription in improving pelvic floor muscle strength and function in postpartum women with FPFD. **Methods**: Thirty postpartum women diagnosed with FPFD underwent a 10-week exercise intervention. This study adopted a single-group pre–post design. Pelvic floor muscle electromyography assessment indicators were measured before and after the intervention. **Results**: The exercise intervention significantly improved the maximum value of fast-twitch muscle fibers (type II) and the average value of slow-twitch muscle fibers (type I) while reducing resting tension and variability. **Conclusions**: A 10-week combined aerobic and resistance training exercise prescription effectively improves pelvic floor muscle strength in postpartum women. It enhances the maximum value of fast-twitch (type II) muscle fibers, reduces rise and recovery times, and improves slow-twitch (type I) muscle fiber function, including increasing the mean value and reducing variability, rise time, and recovery time.

## 1. Introduction

The aging population in China is currently on the rise, while the anticipated increase in birth rates has not been realized. Consequently, China has implemented further adjustments to its fertility policies, leading to a significant surge in childbirth since the relaxation of the one-child policy. As a result, there has been an increasing prevalence of pelvic floor dysfunction among women [1,2], which has emerged as a prominent factor impacting their physical and mental well-being.

Female pelvic floor dysfunction (FPFD) is primarily attributed to connective tissue or ligament damage that compromises the support of the pelvic floor [3]. This non-life-threatening condition significantly impacts women’s quality of life. The muscles comprising the pelvic floor are not a singular, cohesive muscle entity [4,5,6,7,8]. Within the human pelvis, there exists a circular arrangement of small muscle groups resembling a hammock, providing support for abdominal organs such as the female urethra, bladder, vagina, uterus, and rectum. Governed by pelvic floor nerves, these muscles contract and relax in order to regulate urination and defecation while also maintaining vaginal tightness, among other physiological functions [9,10].

The high-risk factors for FPFD include pregnancy and childbirth, which can be regarded as prevalent chronic conditions among women. With the increasing number of multiparous women in our country, the incidence of FPFD is also expected to rise [11,12,13,14,15]. Enhancing pelvic floor rehabilitation management, prevention, and treatment for pregnant and postpartum women has become a significant societal concern. Pregnancy and childbirth impact the pelvic floor function of postpartum women, making the late pregnancy period and postpartum period an opportune window for preventing and treating FPFD [16,17]. Existing therapies for pelvic floor dysfunction include surgical interventions and traditional physical therapy. However, these methods can be invasive and costly. Exercise therapy, on the other hand, offers a non-invasive and cost-effective alternative. It not only serves as a treatment modality but also as a preventive measure for pelvic floor dysfunction.

The exercise prescription is a carefully planned therapeutic regimen of physical movements, postures, or activities that aims to facilitate patients in recovering from or preventing injuries, improving functionality, reducing risks, optimizing overall health, and promoting well-being [6,18,19,20,21,22,23,24]. Unlike conventional exercise plans, an exercise prescription program is developed by professional rehabilitation personnel with the objective of attaining specific, measurable outcomes [25]. For female pelvic floor dysfunction, exercise is not only a treatment method but also an important means of preventing such diseases [26]. This study aims to explore the problem of female pelvic floor dysfunction through the formulation of exercise prescriptions [27], effectively alleviating the discomfort caused by pelvic muscle dysfunction.

Based on the literature, we hypothesize that combined aerobic and resistance training will significantly improve the strength and endurance of both fast-twitch and slow-twitch pelvic floor muscles in postpartum women. The following hypotheses are proposed for this study:

1. Aerobic exercise can enhance the subjects’ slow-twitch muscle fiber’s strength and stability, while resistance training strengthens their fast-twitch muscle fibers. Both types of exercises coordinate with each other and promote each other.
2. A 10-week combined aerobic and resistance training exercise prescription will significantly improve the strength and endurance of both fast-twitch and slow-twitch pelvic floor muscles in postpartum women.
3. This exercise prescription will effectively enhance the fast muscle strength and reaction speed, as well as the endurance of slow muscles and the stability of contraction control, thereby alleviating the symptoms of pelvic floor dysfunction.

Female pelvic floor dysfunction (FPFD) is primarily attributed to connective tissue or ligament damage that compromises the support of the pelvic floor. This non-life-threatening condition significantly impacts women’s quality of life. Conservative treatments, such as pelvic floor muscle training (PFMT), have shown promise in managing FPFD. However, the effectiveness of combined aerobic and resistance training in this context remains underexplored. Recent studies highlight the efficacy of structured exercise regimens for FPFD. For instance, Navarro-Brazález et al. [7] found hypo-pressive exercises effective in reducing pelvic floor dysfunction symptoms. Similarly, Radziminska et al. [11] demonstrated that pelvic floor muscle training (PFMT) significantly improves urinary incontinence and quality of life in women with FPFD. These findings align with our hypothesis that a combined aerobic-resistance training protocol may synergistically enhance pelvic floor muscle function by targeting both slow- and fast-twitch fibers.

Understanding the effects of exercise prescription on slow-twitch (type I) and fast-twitch (type II) muscle fibers is necessary for establishing optimal intervention measures. Type I (slow-twitch) muscle fibers are characterized by slow contraction speed, lower strength, and high endurance, while type II (fast-twitch) muscle fibers are designed for quick actions with higher strength and lower endurance. Assessing both types of fibers is crucial for a comprehensive understanding of pelvic floor muscle function. The main objective of this study is to evaluate the effectiveness of a 10-week combined aerobic and resistance training exercise prescription in improving pelvic floor muscle strength and function in postpartum women with FPFD.

## 2. Materials and Methods

### 2.1. Study Design and Participants

Participants were recruited from the Postpartum Rehabilitation Department of Chizhou People’s Hospital, Anhui Province, from July 2022 to September 2022. The study was advertised through hospital posters and flyers. Interested participants were screened by experienced obstetricians and gynecologists to ensure they met the inclusion criteria. Written informed consent was obtained from all participants.

This study adopted a single-group pre-post design. The pre- and post-intervention physical states of the participants were compared to assess the impact of the 10-week exercise prescription intervention on postpartum women with pelvic floor dysfunction. In the initial stages of the study, we recognized that establishing a control group would have provided additional insights. However, due to limited resources and the difficulty in recruiting a sufficient number of participants within the study period, we decided to focus on a single-group design. Future research could expand on this study by incorporating a control group to further validate the effectiveness of the exercise prescription.

### 2.2. Subjects

Participants were recruited through postpartum clinics at Chizhou People’s Hospital. A priori sample size calculation using G*Power 3.1.9.7 (α = 0.05, β = 0.20, effect size d = 0.6) yielded a required *n* = 21. To account for potential attrition and non-completion of the 10-week exercise rehabilitation program, 30 participants were enrolled. The participants’ ages ranged from 22 to 35 years old. Prior to commencing the experiment, individual consultations were conducted with each participant to alleviate their psychological pressure. Additionally, relevant questionnaires were administered to collect information on their physical and psychological conditions after childbirth for basic investigation purposes. A validated 10-item questionnaire adapted from the Pelvic Floor Distress Inventory (PFDI-20) was administered to assess physical symptoms (e.g., urinary incontinence, pelvic pain) and psychological distress (e.g., anxiety, social avoidance) on a 5-point Likert scale. The participants were fully informed about the experimental procedures and potential effects, following which they provided written informed consent along with a personal privacy confidentiality agreement. The specific selection criteria for postpartum women are as follows.

#### 2.2.1. Inclusion Criteria

(1) Include primiparous women aged between 22 and 35 who have undergone vaginal deliveries, had healthy newborns, and engaged in breastfeeding after childbirth.
(2) Include women who have had vaginal deliveries within the time frame of 42 days to 3 months postpartum.
(3) The training period is set at ten weeks; participants need to ensure they conduct pelvic floor muscle tests both before and after the experiment for assessment purposes.

#### 2.2.2. Exclusion Criteria

(1) Firstly, exclude postpartum women with chronic physical illnesses, mental disorders, severe liver disease in conjunction with pregnancy, severe psychological disorders, or a history of significant illness (autoimmune disorders or neurological impairments).
(2) Exclude women during the puerperal period (within 42 days after childbirth), as well as those between 3 and 12 months postpartum.

### 2.3. Procedure

The exercise intervention program in this study utilized a modular exercise prescription comprising low-intensity aerobic training combined with resistance training. No control group was established, and the pre- and post-intervention physical states were compared to emphasize the experimental effects. Prior to the exercise intervention, participating mothers commence with warm-up exercises followed by 30 min of resistance training in the rehabilitation exercise equipment room. Subsequently, they took a 5-min rest before proceeding to aerobic exercises. Muscle relaxation stretching was performed upon completion of the exercises. The stretching exercises included gentle stretches for the pelvic floor muscles, aiming to improve muscle flexibility and reduce tension. Specific stretching exercises included the following: Supine Pelvic Tilt; Knee-to-Chest Stretch; and Side-Lying Leg Lift. All relevant exercise prescriptions were conducted under one-on-one guidance from experiment personnel in the hospital’s rehabilitation exercise training room, ensuring optimal physical and mental well-being for mothers. Exercise adherence and compliance were monitored through a combination of methods: attendance records, exercise diaries, and regular check-ins.

**Figure 1:**
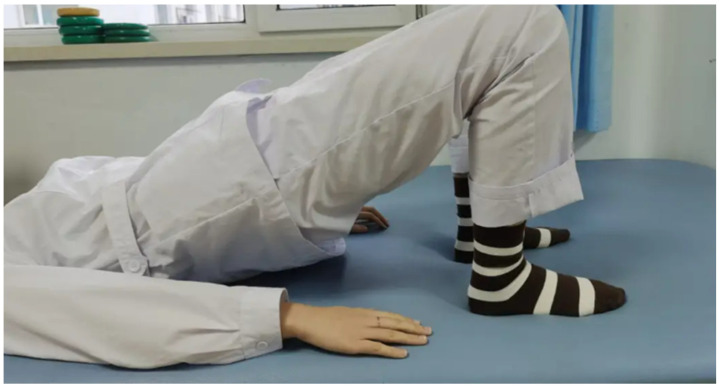
Schematic diagram of bridge hip-raises.

The 10-week exercise prescription was designed based on the characteristics of pelvic floor muscle fibers and the principles of exercise rehabilitation. The prescription was designed by a panel of pelvic floor physiotherapists and aligned with the American College of Sports Medicine (ACSM) guidelines for postpartum populations. For resistance training, we chose Kegel exercises and bridge hip-raises. Kegel exercises are classic for pelvic floor muscle training. In the first 2 weeks, participants were required to perform Kegel exercises 3 times per day, with each session consisting of 10 slow contractions (hold for 5–6 s each) and 10 fast contractions (contract and relax quickly). Starting from the 3rd week, the number of slow contractions was increased to 15, and the hold time was extended to 8 s. For bridge hip-raises, as shown in Figure 1, in the first 2 weeks, participants completed 2 sets of 10 repetitions, 2 times per day. From the 3rd week, the number of sets was increased to 3, and the number of repetitions to 12.

For aerobic training, we selected cycling and brisk walking. In the first 2 weeks, participants cycled on a stationary bike for 20 min at a low-intensity level (maintaining a heart rate at about 60–70% of their maximum heart rate) 3 times per week. They also completed brisk walking for 20 min, 3 times per week. From the 3rd week, the cycling time was increased to 25 min, and the intensity was adjusted to maintain a heart rate at 70–80% of the maximum heart rate. The brisk-walking time was also increased to 25 min. As the weeks progressed, in the 7th–10th weeks, the cycling time was further extended to 30 min, and the brisk-walking time remained at 25 min. The intensity of both cycling and brisk walking was maintained at an appropriate level to ensure a good aerobic exercise effect while avoiding over-fatigue. All the exercise intensities and durations were adjusted according to the participants’ physical conditions and feedback during the training process. This combination of aerobic and resistance training was designed to comprehensively improve the strength, endurance, and function of pelvic floor muscles, with a scientific basis in the different effects of aerobic and resistance exercises on slow-twitch and fast-twitch muscle fibers.

The comprehensive score and individual scores obtained from the pelvic floor electrocardiograph assessment serve as crucial observational indicators for postpartum women undergoing exercise intervention. Two tests are conducted before and after the intervention using the PHENIX USB 4.0 pelvic floor muscle testing device. The PHENIX USB 4.0 pelvic floor muscle testing device (SANSAN, Paris, France) was used for electromyography. This device records surface electromyography (sEMG) signals at a sampling rate of 1000 Hz, with validated clinical reliability (intraclass correlation coefficient > 0.85). Parameters included resting tone (μV), fast/slow-twitch fiber activation, and variability (coefficient of variation). Assessments were conducted at baseline and after the 10-week intervention using the PHENIX USB 4.0 pelvic floor muscle testing device. The primary variables measured were the maximum value of fast-twitch muscle fibers and the average value of slow-twitch muscle fibers. Secondary variables included resting tension and variability.

The study initially obtained the comprehensive score of pelvic floor muscles in postpartum women, which serves as a crucial reference indicator and an essential comparative measure for experimental outcomes. If participants’ total score falls below 60 points, they are required to undergo pelvic floor rehabilitation treatment, typically involving 2–3 courses. For those with a total score ranging from 60 to 80 points, it is recommended that postpartum women receive pelvic floor rehabilitation treatment, usually requiring 1–2 courses. If the total score reaches or exceeds 80 points, it is considered satisfactory and can be enhanced and maintained through voluntary participation in daily Kegel exercises. This experiment does not involve a control group. Comparative results of the experiment are based on both overall scores and sub-scores before and after physical intervention for each participant.

To perform manual detection, a disposable isolation sheet is placed on the treatment bed. One side of the patient’s pants is pulled down to expose their external genitalia, and they assume a semi-sitting or lying position with knees apart. Meanwhile, the examiner lightly presses their left palm on the patient’s abdomen and slowly inserts their right middle finger and index finger into the patient’s vagina to initiate testing. The manual detection method was justified based on its reliability and validity in previous studies [2,3]. Inter-rater reliability was assessed by having two independent assessors evaluate a subset of participants (*n* = 10). The inter-rater reliability was found to be high, with an intraclass correlation coefficient (ICC) of 0.92, indicating excellent agreement between assessors. This method was chosen for its practicality and accuracy in assessing pelvic floor muscle function in a clinical setting.

During testing, patients are instructed to contract their vaginal muscles upon command. The contraction duration and number of consecutive completions are graded accordingly. Patients should try not to engage their abdominal muscles during vaginal contractions in order to differentiate between abdominal muscle contractions and anal sphincter contractions.

The comprehensive score and individual indicators obtained from the assessment of pelvic floor muscle electromyography are as follows:

(1) Assessment during rest phase: There are primarily two indicators, namely the mean value and variability. If the mean value exceeds 4 μV, it indicates an elevation in resting tension of the pelvic floor muscles, commonly referred to as high tone. The pelvic floor muscles remain in a tense and hyperactive state, resulting in ischemia/hypoxia of the pelvic floor muscles, characterized by dyspareunia, urinary retention, bladder pain, constipation, and vulvar pain; furthermore, the pelvic floor muscles are more susceptible to fatigue, which can impact our daily exercise regimen. If the variability is greater than or equal to 0.2, it may be accompanied by issues such as pelvic pain.
(2) Evaluation of fast-twitch muscle testing: There are three primary indicators, namely maximum value, rise time, and recovery time. If the maximum value falls below the reference range, it indicates weakened strength of fast-twitch muscles in the pelvic floor, which can contribute to stress urinary incontinence, fecal incontinence, orgasmic dysfunction, and sexual frigidity. In cases where the rise time exceeds or equals 0.5 s, it is often accompanied by postpartum urinary incontinence and pelvic organ prolapse. Similarly, if the recovery time surpasses or equals 0.5 s, it is commonly observed in individuals with chronic pelvic pain.
(3) Assessment of slow-twitch muscle: There are two primary data points, namely the mean value and the degree of variability. If the mean value falls below the reference range, it indicates insufficient strength and weakness in slow-twitch muscles. The main concern associated with weak slow-twitch muscle strength is its potential to contribute to pelvic organ prolapse, such as uterine prolapse and vaginal wall bulging. Furthermore, it may also give rise to issues like bowel dysfunction, vaginal laxity, sexual frigidity, and recurrent urinary reproductive tract infections. A variability equal to or exceeding 0.2 signifies a reduction in pelvic floor muscle stability commonly observed in postpartum urinary leakage problems.
(4) Evaluation of the endurance testing phase: Two key data points are considered, namely the mean value and variability. Endurance testing also evaluates slow twitch muscles and only yields relevant test results following exercise intervention. A decrease in the ratio of mean value to the last 10 s compared to the first 10 s indicates inadequate endurance of slow twitch muscles, which can contribute to pelvic pain and vaginal laxity issues. If variability exceeds 0.2, it signifies a decline in pelvic floor muscle stability, commonly observed in postpartum urinary leakage problems.
(5) Evaluation during the resting phase: There are two primary data points, namely the mean value and variability. If the mean value exceeds 4 μV, it indicates an elevation in pelvic floor muscle resting tension, which can result in clinical symptoms such as dyspareunia, urinary retention, and constipation due to pelvic floor muscle ischemia. In case the variability of the data surpasses the reference range, there may be a potential issue related to chronic pelvic pain.

### 2.4. Statistical Analyses

The measured data in this article were processed using EXCEL 2021 software for statistical analysis, both before and after the experiment. The experimental data were represented by mean standard deviation (M ± SD) and analyzed using SPSS 26.0. Following a normality test, a paired sample *t*-test was employed to compare the pre- and post-experiment results, with *p* < 0.05 considered statistically significant and *p* < 0.01 considered highly significant. When *p* > 0.05, it is generally regarded as lacking statistical significance. Data normality was confirmed using Shapiro–Wilk tests (*p* > 0.05). Paired *t*-tests were applied for normally distributed data; non-parametric Wilcoxon signed-rank tests were used for skewed variables.

## 3. Results

### 3.1. Baseline Characteristics

**Figure 2:**
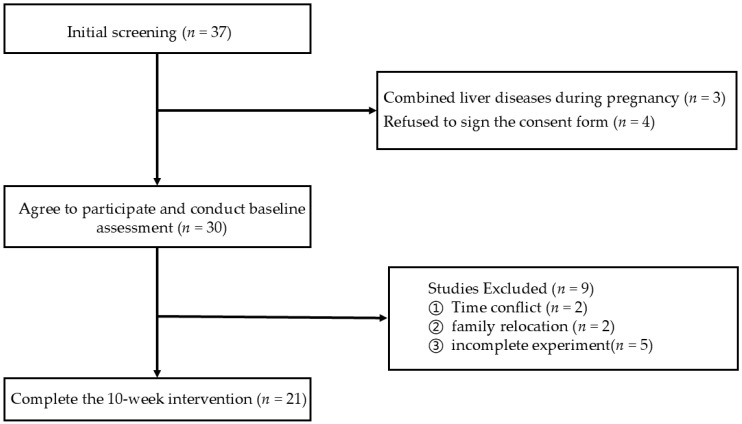
Flowchart of participant recruitment, allocation, and completion.

Figure 2 is a flowchart of participant recruitment, allocation, and completion, following the CONSORT guidelines. A total of 30 participants were initially enrolled in this experiment. Two participants withdrew due to scheduling conflicts, two due to family relocation, and five failed to complete the 10-week exercise rehabilitation program due to physical reasons, resulting in 21 completers.

Table 1 presents the basic characteristics of 21 study participants, providing insights into their physical and demographic attributes. In terms of physical traits, the average height of the participants is 161.72 cm with a standard deviation of 3.5 cm, indicating a relatively narrow range of heights within the group. The mean weight is 58.39 kg, with a standard deviation of 4.2 kg, also suggesting limited variation among individuals. The average BMI of 22.76 kg/m^2^, with a standard deviation of 2.0, falls within a normal range, which may be relevant when considering the impact of the intervention on postpartum women with pelvic floor dysfunction, as body composition can influence pelvic floor muscle function.

Regarding demographic factors, the educational level distribution shows that 6 participants have a high-school education or below, while 15 have a bachelor’s degree or above. This disparity might influence how participants understand and adhere to the exercise program. In terms of occupation, 8 are engaged in light-physical work and 13 in heavy-physical work. Different work types may affect the participants’ physical condition and available time for the exercise intervention. The family income level is split with 10 in the medium-income category and 11 in the high-income category. A higher income may provide more resources and time for recovery, which could potentially impact the outcomes of pelvic floor rehabilitation.

### 3.2. Comprehensive and Sub-Item Indicators of the Subjects

This study aimed to evaluate the effectiveness of a 10-week exercise prescription on pelvic floor muscle function in postpartum women. The results were assessed by comparing pelvic floor muscle function scores and sub-item scores before and after the exercise intervention. As presented in Table 2, the overall score of pelvic floor muscle function showed a significant improvement after the exercise intervention. The mean score increased from 51.129 ± 16.86 before the intervention to 73.481 ± 9.642 after the intervention, with a *p*-value of less than 0.01. This indicates a statistically significant and clinically meaningful improvement in pelvic floor muscle function.

Specifically, the scores for fast-twitch muscle fibers (type II) increased significantly from 56.71 ± 26.443 to 78.05 ± 14.647 (*p* < 0.01), demonstrating improved strength and reaction speed. Similarly, the scores for slow-twitch muscle fibers (type I) showed a significant increase from 35.33 ± 23.081 to 68.38 ± 17.721 (*p* < 0.01), indicating enhanced endurance and stability. Although the pre-resting and post-resting stage scores showed slight improvements, these changes were not statistically significant (*p* = 0.157 and *p* = 0.885, respectively). These results suggest that the exercise prescription effectively enhances pelvic floor muscle function, particularly in improving fast-twitch muscle strength and slow-twitch muscle endurance, making it a valuable intervention for postpartum pelvic floor dysfunction.

The 10-week exercise prescription significantly improved pelvic floor muscle function in postpartum women, as evidenced by the significant increase in the overall score and the significant improvements in the sub-item scores for fast-twitch and slow-twitch muscle fibers. These results suggest that the exercise prescription is effective in enhancing pelvic floor muscle strength and function, particularly in improving the strength of fast-twitch muscle fibers and the endurance and stability of slow-twitch muscle fibers. The lack of significant improvement in the pre-resting and post-resting stages may indicate that these aspects of pelvic floor function are less responsive to the exercise intervention or may require a longer duration of training to show significant changes.

### 3.3. The Electromyographic Evaluation Indicators of Subjects at Various Stages

The pre-resting phase primarily assesses the static tension of the pelvic floor muscles prior to exercise, which reflects their relaxation function. If the value exceeds the reference range, it indicates an elevated resting tension in the pelvic floor muscles for a duration of one minute. When all test values surpass the reference range, it suggests excessive muscle tension where complete relaxation of the pelvic floor muscles is hindered, resulting in fatigue and pelvic pain. In such cases, it is essential to avoid excessive exercises targeting the pelvic floor muscles and first ensure their relaxation before engaging in physical activity. The electrocardiograph evaluation during the pre-resting phase includes two main indicators: average value and variability. The evaluation criteria for subjects during this phase are presented in Table 3. Based on the *p*-values provided in Table 3, we can conclude that there exists a significant difference (*p* < 0.05) in average value between pre- and post-intervention for pregnant women; furthermore, there is also a highly significant difference (*p* < 0.01) in variability between pre- and post-intervention during this phase.

The post-resting phase refers to the assessment of static tension in the pelvic floor muscles, where a value greater than 4 μV indicates an elevation in resting tension. The judgment method for the post-resting phase is identical to that used for the pre-resting phase. Some data suggest variability beyond reference values, indicating potential issues related to chronic pelvic pain. Table 3 presents electromyographic evaluation indicators during the post-resting phase of subjects. Based on the *p*-values provided in Table 3, it can be concluded that there is no significant difference in both average value and variability during the post-resting phase before and after exercise intervention for parturient (*p* > 0.05).

Table 3 above presents the effect sizes of the exercise prescription intervention on various outcomes related to pelvic floor dysfunction in postpartum women. The effect sizes were calculated using Cohen’s d, which provides a measure of the standardized difference between the pre-intervention and post-intervention means. The interpretations are based on Cohen’s guidelines, where an effect size of 0.2 is considered small, 0.5 is medium, 0.8 is large, and greater than 1.0 is very large.

The exercise prescription intervention demonstrated significant improvements in pelvic floor muscle function in postpartum women, as evidenced by the effect sizes calculated using Cohen’s d. The absolute effect sizes were used to interpret the practical significance of the findings. The intervention led to a large reduction in the average value and variability of the pre-resting stage, indicating a significant decrease in resting tension and improved consistency of muscle relaxation. Additionally, there was a large increase in the maximum value of fast-twitch muscle fibers and a very large increase in the average value of slow-twitch muscle fibers, suggesting enhanced strength and endurance of these muscle fibers. The intervention also resulted in moderate to large reductions in the ascending and recovery times of both fast-twitch and slow-twitch muscle fibers, indicating improved muscle contraction and relaxation speeds. However, the effect sizes for the post-resting stage were small, indicating minor improvements in resting tension and variability. Overall, the exercise prescription intervention was effective in enhancing pelvic floor muscle function, with significant improvements observed in key outcomes such as resting tension, muscle strength, and endurance.

Fast-twitch (type II muscle fibers) phase: This phase primarily assesses the muscular strength and reaction speed of dynamic fast-twitch (type II muscle fibers). Insufficient strength in these muscles can result in stress urinary incontinence, fecal incontinence, decreased sexual desire, and diminished sexual experience. The electrical evaluation of fast-twitch muscles encompasses three main indicators: maximum value, rise time, and recovery time. If the maximum value is below the reference value, it indicates weak pelvic floor fast-twitch muscle strength, which may lead to stress urinary incontinence, fecal incontinence, orgasmic dysfunction, and reduced sexual desire. A rise time equal to or greater than 0.5 s often accompanies postpartum urinary incontinence and pelvic organ prolapse. Recovery time equal to or greater than 0.5 s is commonly observed in chronic pelvic pain. The electromyographic evaluation indicators for the fast-twitch (type II muscle fibers) phase during this stage are presented in Table 3. Based on the *p*-values provided in Table 3, it can be concluded that there is a highly significant difference (*p* < 0.01) between pre- and post-intervention values for maximum value during the fast-twitch (type II muscle fibers) phase among the participants; however, no significant difference was observed for rise time and recovery time before and after exercise intervention among the participants (*p* > 0.05).

During the slow-twitch (type I muscle fibers) stage, the primary focus is on assessing dynamic slow-twitch (type I muscle fibers) strength and contraction control stability. A decrease in strength and an increase in variability greater than 0.2 can lead to stress urinary incontinence, pelvic organ prolapses, and bowel dysfunction. The slow-twitch stage includes four data points: mean value, variability, rise time, and recovery time. If the mean value falls below the reference value, it indicates insufficient slow-twitch muscle strength or weakness, with the main concern being pelvic organ prolapse caused by weak slow-twitch muscles. Variability equal to or greater than 0.2 suggests decreased pelvic floor muscle stability commonly seen in postpartum urinary leakage issues. The endurance testing stage evaluates pelvic floor muscle endurance with a particular emphasis on slow-twitch muscles. A decline in mean value and posterior-to-anterior ratio indicates reduced slow-twitch muscle endurance, while exercise intervention yields corresponding results for measuring these muscles’ performance levels only after completion of testing procedures. Poorer endurance of slow-twitch muscles reflected by lower ratios between posterior 10 s and anterior 10 s, as well as decreasing mean values, may result in fatigue-related pelvic pain and vaginal laxity.

The electrocardiograph evaluation indicators of the slow-twitch muscle fibers (type I fibers) in the subjects are presented in Table 3. Based on the *p*-values provided in Table 3, it can be inferred that there exists a highly significant difference (*p* < 0.01) in both the mean and variability of the slow-twitch muscle fiber stage before and after exercise intervention for pregnant women, whereas a significant difference (*p* < 0.05) is observed in both the rise time and recovery time of slow-twitch muscle fiber stage before and after exercise intervention for pregnant women.

### 3.4. Experimental Analysis of Pelvic Floor Distress Inventory-20 (PFDI-20) Scores

A validated 10-item questionnaire adapted from the Pelvic Floor Distress Inventory (PFDI-20) was administered to assess physical symptoms and psychological distress on a 5-point Likert scale. Baseline results in Table 4 indicated mean scores of 45.2 ± 12.3 for physical symptoms and 38.7 ± 10.8 for psychological distress, which were later correlated with post-intervention improvements in pelvic floor metrics.

The mean score for physical symptoms significantly decreased from 45.2 to 30.5 after the intervention, indicating a substantial improvement in physical symptoms related to pelvic floor dysfunction. The reduction in the standard deviation suggests a more consistent improvement across participants. The mean score for psychological distress also showed a significant decrease from 38.7 to 25.3, reflecting a notable reduction in psychological distress experienced by the participants. The decrease in the standard deviation indicates a more uniform improvement in psychological well-being.

The intervention led to significant improvements in both physical symptoms and psychological distress, as evidenced by the substantial reductions in mean scores and the more consistent improvements indicated by the reduced standard deviations. These results suggest that the intervention was effective in addressing pelvic floor dysfunction and related psychological issues.

## 4. Discussion

### 4.1. The Impact of Exercise Prescription on FPFD During the Pre- and Post-Resting Stages

After a 10-week intervention combining aerobic and resistance exercises, there was a significant difference in the mean value of the pre-resting stage for postpartum women before and after the exercise intervention (*p* < 0.05). The variability of the pre-resting stage also exhibited a highly significant difference before and after the exercise intervention (*p* < 0.01). However, there were no significant differences in the mean value and variability of the post-resting stage before and after the exercise intervention (*p* > 0.05). Following the implementation of an exercise prescription, there was some improvement in the pre-resting stage for postpartum women but no noticeable change in the post-resting stage after exercise intervention. Based on experimental test results from this study, it can be concluded that following a comprehensive 10-week systematic exercise prescription treatment, there was a statistically significant decrease of 36.2% in average values during the pre-resting stage from 4.411 μV to 2.812 μV (*p* < 0.05), as well as a highly significant decrease of 37.0% in variability during the pre-resting stage from 0.276 to 0.174 (*p* < 0.01) after exercise intervention. The majority of assessed postpartum women had average values below 4 μV during their pre-resting stages and variability below 0.2, indicating successful achievement with this exercise prescription.

After 10 weeks of comprehensive exercise prescription treatment, the average value of the post-resting stage decreased from 4.477 μV to 4.063 μV; however, it still remained above the threshold of 4 μV, indicating that a majority of postpartum women exhibited elevated resting tension in their pelvic floor muscles. The variability of the post-resting stage after exercise intervention also showed improvement, decreasing from 0.185 to 0.166. Nevertheless, no statistically significant difference was observed in both the mean value and variability of the post-resting stage between pre- and post-exercise intervention among the participants (*p* > 0.05).

### 4.2. The Effects of Exercise Prescription on the Fast-Twitch and Slow-Twitch Muscle Fibers

The experimental test results of this study led to the conclusion that, following a 10-week comprehensive exercise prescription treatment, there was a significant increase in the maximum value of fast-twitch muscle fibers (type II fibers) from 26.91 μV to 40.73 μV, representing a remarkable improvement of 36.2% (*p* < 0.01). Moreover, the rise time of fast-twitch muscle fibers in the exercise intervention group decreased from 0.523 to 0.442, indicating a reduction of 37%. Similarly, the recovery time also decreased from 0.751 to 0.515, reflecting a decrease of 37%. However, it is worth noting that these changes did not reach statistical significance (*p* > 0.05). Nonetheless, this experiment demonstrated that exercise intervention yielded certain effects, which are generally consistent with previous research findings.

After the exercise intervention, there was a significant 81.33% improvement in the average value of slow-twitch muscle fibers (type I muscle fibers), which increased from 16.741 μV to 30.357 μV (*p* < 0.01). The variability of slow-twitch muscle fibers also significantly decreased by 30.72%, from 0.319 to 0.221 after exercise intervention (*p* < 0.01). Additionally, the rise time of slow-twitch muscle fibers decreased by 34.22%, from 0.713 to 0.469, and the recovery time for slow-twitch muscles reduced by an impressive 51.83%, from 1.646 to 0.793 (both *p* < 0.05). Overall, aerobic exercise comprehensively enhanced the strength of participants’ slow-twitch muscles, contributing to pelvic organ prolapse prevention and improved muscular stability.

Our findings align with Bø et al. [16], who reported a 30% improvement in pelvic floor strength after 12 weeks of PFMT. However, unlike Kashanian et al. [12], who focused solely on Kegel exercises, our combined aerobic-resistance approach yielded greater enhancements in type II fiber activation (40.73 μV vs. 26.91 μV, *p* < 0.01), suggesting synergistic benefits. This novel integration addresses both endurance (type I fibers) and rapid contraction (type II fibers) needs, advancing current rehabilitation paradigms by offering a holistic solution for FPFD. Despite high variability in recovery time (e.g., 0.751 ± 0.772 pre-intervention), the observed reductions post-intervention (0.515 ± 0.156) reflect clinically meaningful improvements, consistent with trends reported by Gagnon et al. [20]. These findings suggest that an exercise regimen integrating aerobic exercise and resistance training exerts a substantial positive influence on pelvic floor dysfunction. More specifically, this intervention significantly decreased the resting tension of the pelvic floor muscles, enhanced the strength and response velocity of fast-twitch muscle fibers, and improved the endurance and stability of slow-twitch muscle fibers. These enhancements provide robust scientific evidence supporting the pelvic floor rehabilitation of postpartum women.

Furthermore, the research also suggests that this exercise prescription significantly enhances the maximum value of fast-twitch (type II) muscle fibers while decreasing ascent time and recovery time. It strengthens fast-twitch muscle strength and reaction speed, thus mitigating urinary or fecal incontinence. Simultaneously, it improves the mean value of slow-twitch (type I) muscle fibers by reducing their variability as well as ascent time and recovery time. This intervention enhances slow-twitch endurance and contraction control stability to prevent pelvic organ prolapse.

The reduction in rise time and recovery time for fast-twitch muscle fibers, although not statistically significant, may still be clinically relevant. These changes suggest a trend toward improvement that could become more pronounced with additional data or a longer intervention period. The observed improvements in rise and recovery time may indicate enhanced neural activation and muscle coordination, which are essential for effective pelvic floor function. Future studies with larger sample sizes and longer intervention periods could further explore these trends.

The significant improvement in slow-twitch muscle fibers (type I) is compelling and can be linked to specific aspects of the aerobic exercise regimen. The endurance training component of the exercise prescription likely contributed to the enhanced stability and control of slow-twitch muscle fibers. These findings are consistent with previous studies demonstrating the positive effects of aerobic exercise on muscle endurance and stability. The improvements observed in this study suggest that combined aerobic and resistance training may be particularly effective in enhancing pelvic floor function in postpartum women.

The characteristics of slow-twitch muscle fibers include a reduced contraction speed, lower strength levels, efficient utilization of oxygen, and enhanced endurance capacity. Slow-twitch muscle fibers are rich in red-colored proteins, such as myoglobin and cytochrome, which contribute to the reddish appearance of muscles. They possess a greater ability to generate energy through aerobic metabolism and exhibit slower nerve conduction velocity. Additionally, slow-twitch muscle fibers have smaller diameters and are less prone to hypertrophy. Training these fibers primarily involves prolonged durations and multiple repetitions until they reach a state of extreme soreness before experiencing any noticeable growth. With sufficient training duration, slow-twitch muscle fibers will undoubtedly undergo significant development.

In conclusion, aerobic exercise enhances the strength and stability of slow-twitch muscles in subjects, while resistance training strengthens their fast-twitch muscle strength. These two forms of exercise complement each other and mutually promote one another. The division of labor between fast-twitch fibers and slow-twitch fibers is a remarkable biological design. During periods of inactivity, humans primarily rely on slow-twitch fibers to counteract gravity, such as maintaining posture while standing, which is more efficient. Fast-twitch fibers are specifically designed for rapid movements. Systematic and regular pelvic floor exercises are an excellent method for increasing pelvic floor muscle strength and improving postpartum female pelvic floor dysfunction. Therefore, combining aerobic exercise with resistance training has a more pronounced alleviating effect on female pelvic floor dysfunction, leading to expected rehabilitation outcomes. The significance of this approach for clinical practice is substantial. Integrating both aerobic and resistance training into postpartum care plans can effectively enhance pelvic floor function and significantly improve the overall quality of life.

### 4.3. Limitations

Limitations include the single-group design, which cannot exclude time-related confounders (e.g., natural postpartum recovery). The small sample size limits generalizability and the lack of long-term follow-up precludes conclusions about sustained benefits. Future studies should consider using a randomized controlled trial (RCT) design to better control for these factors and provide more robust evidence for the effectiveness of the exercise prescription.

## 5. Conclusions

The study indicates that a 10-week exercise prescription, which combines aerobic exercise and resistance training, effectively reduces the mean value and variability of the resting phase. This intervention prevents pelvic floor muscles from remaining in a tense and overactive state, thereby alleviating muscle fatigue and reducing the likelihood of pelvic floor muscle ischemia/hypoxia. In this study, aerobic exercise was found to effectively enhance the endurance of slow-twitch muscles in the pelvic floor, while resistance training demonstrated its efficacy in strengthening fast-twitch muscles. The 10-week exercise prescription effectively enhanced pelvic floor muscle strength and function in postpartum women. Future studies should include larger sample sizes and control groups to further validate these findings.

## Figures and Tables

**Table 1:** Basic characteristics of the study participants.

Participant Characteristics	Mean ± SD or Frequency	Number of Participants
Height (cm)	161.72 (3.5)	21
Weight (kg)	58.39 (4.2)	21
BMI (kg/m^2^)	22.76 (2.0)	21
Educational Level (High School or below/Bachelor’s Degree or above)	6/15	21
Occupation (Light-physical/Heavy-physical)	8/13	21
Family Income Level (Medium/High)	10/11	21

**Table 2:** Comparison of pelvic floor muscle function scores and sub-item scores before and after exercise intervention in postpartum women.

Evaluation Metrics	Exercise Prescription		*p*-Value
	After (*n* = 21)	Before (*n* = 21)	
Overall score	73.481 ± 9.642	51.129 ± 16.86	<0.01
Sub-item scores of the pre-resting stage	77.57 ± 15.43	71.71 ± 19.9	0.157
Sub-item scores of fast-twitch muscle fiber (type II muscle fibers) stage	78.05 ± 14.647	56.71 ± 26.443	<0.01
Sub-item scores of slow-twitch muscle fiber (type I muscle fibers) stage	68.38 ± 17.721	35.33 ± 23.081	<0.01
Sub-item scores of the post-resting stage	71.14 ± 24.67	70.48 ± 23.41	0.885

**Table 3:** The electromyographic evaluation indicators of subjects at various stages.

Assessment of Different Stages	Exercise Prescription		Absolute Effect Size (Cohen’s d)	*p*-Value
	After (*n* = 21)	Before (*n* = 21)		
**Pre-resting stage**				0.157
Average value	2.812 ± 2.06	4.41 ± 2.81	0.65	0.012
Variability	0.174 ± 0.156	0.276 ± 0.27	0.46	0.004
**Fast-twitch muscle fiber stage**				<0.01
Maximum value	40.73 ± 11.95	26.91 ± 14.03	1.06	<0.01
Ascending time	0.442 ± 0.145	0.523 ± 0.357	0.23	0.363
Recovery time	0.515 ± 0.156	0.751 ± 0.772	0.31	0.188
**Slow-twitch muscle fiber stage**				<0.01
Average value	30.357 ± 9.366	16.741 ± 8.404	1.53	<0.01
Variability	0.221 ± 0.099	0.319 ± 0.107	0.95	<0.01
Ascending time	0.469 ± 0.171	0.713 ± 0.461	0.58	0.033
Recovery time	0.793 ± 0.214	1.646 ± 1.108	0.75	0.020
**Post-resting stage**				0.885
Average value	4.063 ± 3.047	4.472 ± 3.639	0.12	0.420
Variability	0.166 ± 0.047	0.185 ± 0.157	0.12	0.576

**Table 4:** Pelvic Floor Distress Inventory-20 (PFDI-20) scores before and after intervention.

Subscale	Baseline	Post-Intervention
Physical Symptoms	45.2 ± 12.3	30.5 ± 10.2
Psychological Distress	38.7 ± 10.8	25.3 ± 9.1

## Data Availability

Data are contained within the article.
